# Trends in the incidence of cancers of the breast and female genital tract in Harare, Zimbabwe 1990–2019

**DOI:** 10.1002/ijc.70436

**Published:** 2026-03-09

**Authors:** Eric Chokunonga, Zvavahera Michael Chirenje, Margaret Borok, Rudo Makunike‐Mutasa, Ntokozo Ndlovu, Justice Mudavanhu, Biying Liu, D. Maxwell Parkin

**Affiliations:** ^1^ Zimbabwe National Cancer Registry Parirenyatwa Group of Hospitals Harare Zimbabwe; ^2^ Department of Obstetrics, Gynaecology and Reproductive Science University of California San Francisco San Francisco California USA; ^3^ Department of Internal Medicine University of Zimbabwe, Faculty of Medicine and Health Sciences Harare Zimbabwe; ^4^ Department of Laboratory Diagnostic and Investigative Sciences University of Zimbabwe, Faculty of Medicine and Health Sciences Harare Zimbabwe; ^5^ Department of Oncology, Medical Physics and Imaging Sciences University of Zimbabwe, Faculty of Medicine and Health Sciences Harare Zimbabwe; ^6^ Department of NCDs Ministry of Health and Child Care Harare Zimbabwe; ^7^ African Cancer Registry Network Oxford UK; ^8^ International Agency for Research on Cancer Lyon France; ^9^ Nuffield Department of Population Health University of Oxford Oxford UK

**Keywords:** breast, cancer, cervix, registry, Zimbabwe

## Abstract

Trends in age standardised incidence rates (ASRs) in the black (African) female population of Harare are reported for five cancers—of the breast and genital tract (cervix and corpus uteri, ovary and vulva) over a 30‐year period. The incidence of cervix cancer is very high (ASR of 73.7 per 10^5^) and has increased at a rate of 1% annually over the period, although remaining stable in the most recent 15 years; the increase involves mainly women born between 1950 and 1970. Breast cancer rates are less than half those of cervix, but the increase has been more dramatic—3% annually—although this seems to involve only women aged over 40. The incidence of ovarian cancer has been constant over the 30 years; there was a small increase in the incidence of cancer of the corpus uteri (1.5% annually) and a more marked one for vulvar cancer (5.6% annually), for which the incidence is relatively high, by global standards. Concurrent with the increasing incidence of cancers of the breast and corpus uteri are notable trends of population risk factors such as rising obesity rates and declining fertility. The elevated burden of cervix and vulvar cancers aligns with patterns observed in populations with high HPV prevalence, though population‐specific data remain limited. The results confirm that cancers associated with increasingly affluent lifestyles (breast and corpus uteri) are increasing, although the incidence of cancer of the cervix—an eminently preventable cancer—remains persistently high and is clearly a priority for cancer control.

## Graphical abstract

What's new?

Cancers of the breast and female genital tract represent more than half of all cancers in women in sub‐Saharan Africa. Here, the authors assessed incidence trends in a female population living in urban sub‐Saharan Africa based on the Zimbabwe National Cancer Registry in Harare over a 30‐year period. The results highlight increasing trends in the incidence of breast, vulva, corpus uteri, and, until recently, cervix cancer. These trends appear to be related to population‐level lifestyle changes (fertility, obesity, and contraception) and exposure to HIV and HPV infections. The findings provide important insights into future trends and guidance for cancer control priorities.

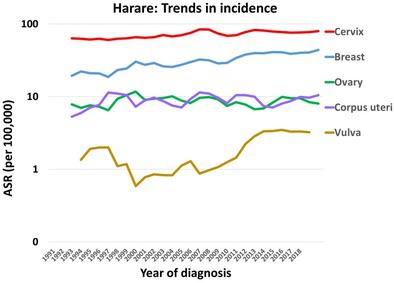

**Abbreviations**
AAPC: average annual percentage change
AIDS: acquired immunodeficiency syndrome
ART: antiretroviral therapy
ASR: age standardised incidence rate
BMI: body mass index
HIV: human immunodeficiency virus
HPV: human papillomavirus
MV: morphological verification
VIA: visual inspection with acetic acid
WLWH: women living with HIV

## 1 INTRODUCTION

Cancers of the breast (ICD 10 C50) and female genital tract (C51–C56) comprise 59% of all cancers of women in sub‐Saharan Africa.^1^ Accordingly, WHO has made the two most common—cancers of the breast and cervix uteri—the focus of special initiatives for their prevention and control.^2^, ^3^ It is clearly essential to possess information on incidence and survival of these important cancers, and how these are evolving over time, in order to plan and evaluate cancer control strategies.

The incidence of cancer of the breast is increasing in Africa, as a consequence of changing lifestyles (diet, fertility, overweight and obesity).^4^ Contrary to what has been observed in most high income countries, the incidence of cancer of the cervix remains high in most of sub‐Saharan Africa.^5^ According to IARC^6^ the risk of cancer of the cervix (and, less certainly, cancer of the vulva) is increased by infection with HIV, so that the epidemic of HIV‐AIDS, which impacted severely on sub Saharan Africa, with the prevalence of HIV infection in adults rising from the 1980's to reach a maximum around 1999–2000,^7^ may have influenced the incidence of these cancers.

In this article, we examine trends in incidence of the major cancers of women in the black population of Harare over a 30‐year period, from 1990 to 2019. The Zimbabwe National Cancer Registry (ZNCR), covering the population of Harare, is one of only two cancer registries in Africa able to document the evolution of cancer patterns over a substantial period (the other being the Kampala Cancer Registry in Uganda). Founded in 1986, it achieved complete coverage of the population of the capital city of Harare in 1990^8^ and the incidence rates for this population have been published in six successive volumes (VII–XII) of “Cancer Incidence in Five Continents” (https://ci5.iarc.who.int/refs).^9^

In a previous paper we presented cancer incidence data from ZNCR for a 20‐year period (1991–2010).^10^ Here we update the latter analysis, including more recent data, focussing on cancers of the female breast and genital tract.

## 2 METHODS

The methods employed by the Zimbabwe National Cancer Registry in Harare have been described previously.^10^, ^11^ Briefly, the registry is situated in the major referral hospital for the northern part of the country (Parirenyatwa Group of Hospitals). It collects information on cancer patients diagnosed and treated in all hospitals and clinics, public and private, as well as pathology laboratories, both by voluntary notification from certain institutions and by staff visits. Medical certification of death (by cause) is relatively complete for the city of Harare, and the registry has used death registrations as an important source of information on cases that may have been missed by the registration process. Death certificate notifications are followed up to obtain additional information on the diagnosis and management of the cancer, and if this proves fruitless, cases are registered based on the death certificate only. Cancer notification forms are filled in for each patient. Information collected includes patient demographic data, as well as details of the tumour, its treatment, the source(s) of information on each case, and follow‐up (date of last contact or death). Information on the abstract forms is coded and entered into the computer using the CanReg5 cancer registration software provided by the IARC. Tumour site and morphology are coded according to the third edition of the International Classification of Diseases for Oncology (ICD‐O).^12^ For tabulation of results, these were converted to the 10th revision of the ICD (ICD 10).

### 2.1 Population

**FIGURE 1:**
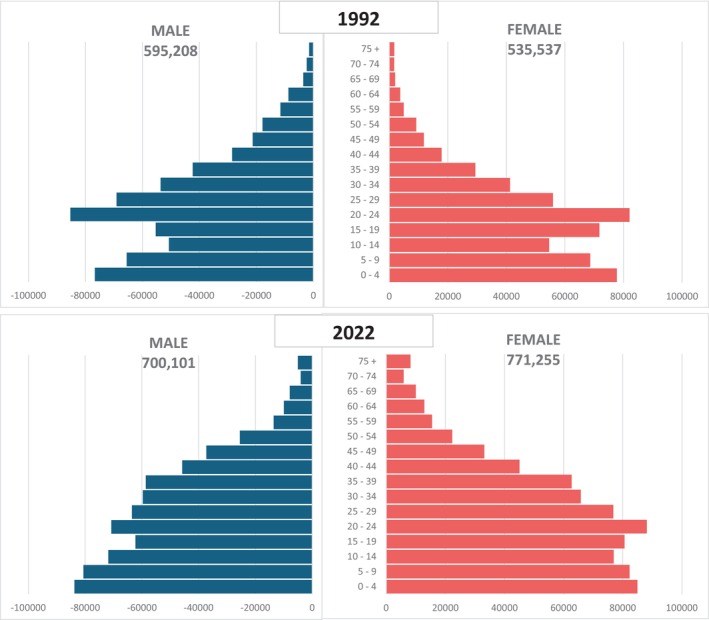
Population pyramids for the black (African) population of Harare (1992 and 2022 census).

Population censuses were performed in 1992, 2002, 2012 and 2022, and for these years, the population of Harare was available by sex, ethnic group and 5‐year age group from ZimStat (the Zimbabwe Statistical Agency). Annual intercensal estimates were prepared, assuming a constant rate of growth within age groups between census counts. Figure 1 shows population pyramids for the black population at the beginning (1992 census) and end (2022 census) of the period studied.

### 2.2 Statistical methods

Incidence rates were calculated for the black population by 5‐year age groups and sex, for each year (1990–2019), and for six 5‐year time periods: 1990–1994, 1995–1999, 2000–2004, 2005–2009, 2010–2014, 2015–2019.

Age standardised rates (ASRs) were calculated using the World Standard Population.^13^ Temporal trends over the whole 30‐year period were examined by fitting a regression line to the log‐transformed age‐standardised incidence rates. From this, we calculated the average annual percentage change (AAPC) as the slope of the regression line, together with its 95% confidence interval.^14^

Two widely used indicators of data quality^15^—the percentage of cases with morphological verification (histology or cytology) of diagnosis (MV%) and the percentage of cases registered solely based on information on a death certificate only (DCO%) were calculated for each sex and the same periods.

## 3 RESULTS

### 3.1 Breast

In the 30 year period studied, 2999 cases of breast cancer were registered (Table 1). The incidence, with an ASR of 42.8 per 100,000 in the most recent decade, is close to the world average of 46.8,^1^ but there has been a brisk increase in incidence over the 30‐year period: 3.0% (95% CI 2.4, 3.7) annually.

**TABLE 1:** Total number of cases registered, age standardised incidence rates (ASRs) in each of the 5‐year periods, and the average annual percentage change (AAPC) in incidence over the period 1990–2019.

Female cancers	ICD 10	Total cases	ASR (95% CI)						AAPC (95% CI), 1990–2019	ASR (95% CI), 1990–2019
			1990–1994	1995–1999	2000–2004	2005–2009	2010–2014	2015–2019		
Breast	C50	2999	21.1	25.8	27.7	32.5	41.5	43.9	3.04 (2.36, 3.73)	33.7 (32.4, 35.1)
Vulva	C51	221	1.4	1.0	0.8	1.0	3.3	3.2	5.56 (1.38, 9.74)	2.1 (1.8, 2.4)
Cervix	C53	6413	63.1	63.0	68.9	75.8	79.8	78.0	1.01 (0.59, 1.42)	73.7 (71.7, 75.7)
Uterus	C54‐55	600	5.9	9.8	8.3	9.8	8.7	10.1	1.52 (0.14, 2.89)	9.2 (8.4, 10.0)
Ovary	C56	760	7.8	9.6	8.7	8.9	8.0	8.5	0.00 (−1.15, 1.16)	8.6 (7.9, 8.3)

**FIGURE 2:**
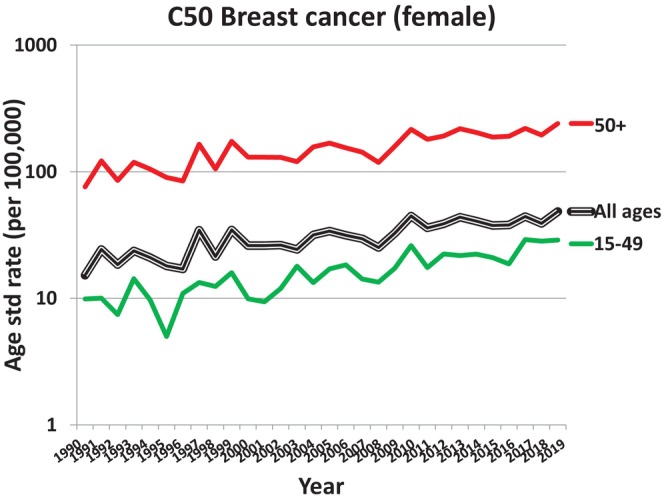
Age standardised rates (per 100,000) for breast cancer in women ages 15–49, 50+, and all ages for the years 1990–2019.

Looking at the trend in pre‐menopausal women (ages 15–49) compared with postmenopausal (aged 50+) the trend is slightly greater in the former (4.0% annually, vs. 3.5%), but the difference is not statistically significant (*p* = .42) (Figure 2).

**FIGURE 3:**
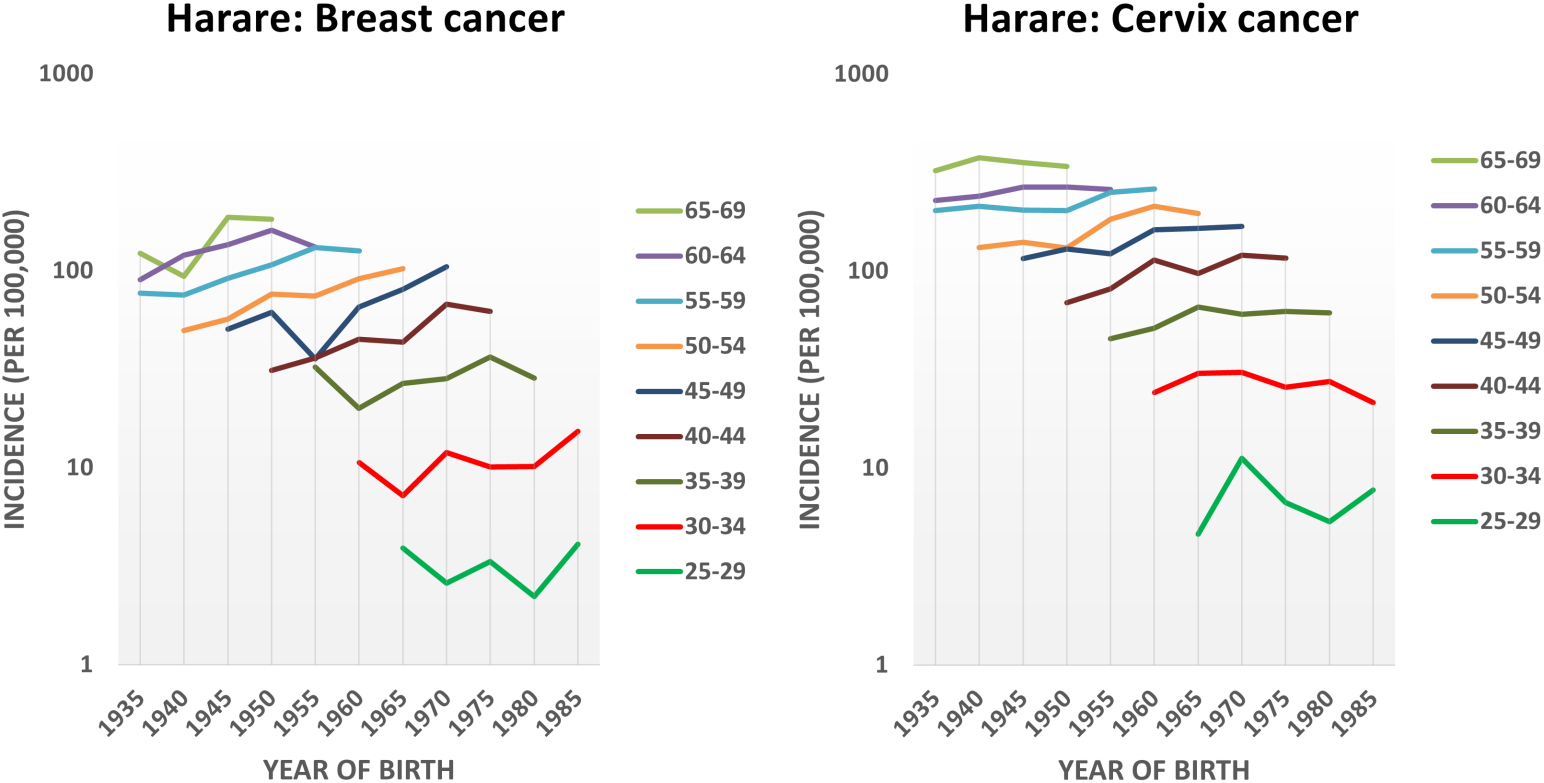
Age specific incidence rates (per 100,000) according to period of birth, for cancers of the breast (left) and cervix uteri (right).

However, looking at trends in incidence according to birth cohort, it does seem that there has been little change in incidence in successive birth cohorts for women under the age of 40, while for older women, incidence increases in successive birth cohorts (Figure 3).

### 3.2 Cervix

The most frequent cancer over the whole period was cancer of the cervix uteri, with 6431 cases registered in the 30‐year period. The incidence in Harare is very high, with an ASR of 78.9 per 100,000 in the most recent decade (2010–2019). Overall, rates have been increasing, the ASR increased at an average annual rate of 1.0% (0.6, 1.4) (Table 1), although there has been almost no change in the second half of the period: AAPC in 2005–2019 was −0.11 (95% CI −1.30, 1.08).

**FIGURE 4:**
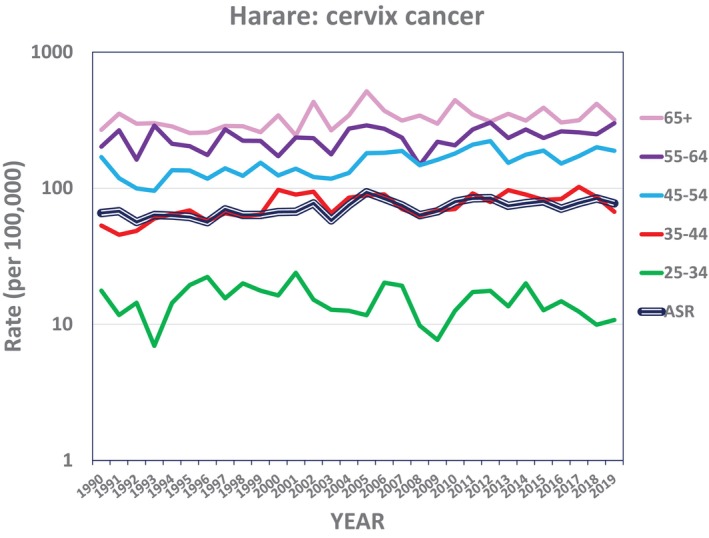
Age specific and age standardised (all ages) incidence rates (per 100,000) for cancer of the cervix uteri (1990–2019).

Examining the trends according to age (Figure 4) shows that rates in the youngest age group (25–34) remained constant, with larger increases (around 1.6% annually) in the middle age range (35–54) and a slower increase (around 0.7% annually) in older women (55+).

Trends by birth cohort suggest that the increases in age specific rates are greatest in women born between 1950 and 1970 (Figure 3), with rather little change in earlier, or more recent, generations.

### 3.3 Ovarian, uterine and vulvar cancers

**FIGURE 5:**
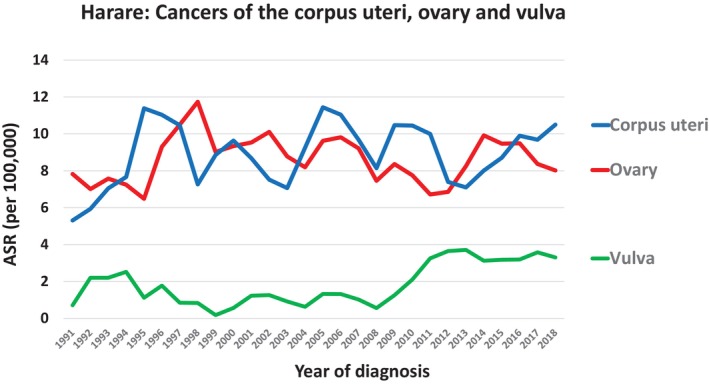
Trends in age standardised rates (per 100,000) for cancers of the ovary, corpus uteri and vulva. Rates are 3‐year moving averages (1991–2018).

Seven hundred and sixty cases of ovarian cancer were registered in the 30‐year period, 600 cases of corpus uteri cancer (we include “Uterus cancer” in this category, since two thirds of the cases recorded as ICD‐10 C55 were aged 50 or above) and 221 cases of vulvar cancer. The age standardised rates in 2020–2019 were 8.3, 9.5 and 3.3 per 100,000 respectively. Figure 5 shows the trends in incidence of these three cancers. A small but statistically significant increase in corpus cancer incidence was observed during the study period (AAPC 1.5%, 95% CI 0.14–2.89). In contrast, ovarian cancer incidence remained stable, with no discernible trend (AAPC 0.0, 95% CI −1.15 to 1.16).

Although the incidence rates of vulvar cancer are low compared with those of the other cancers considered, the trend in incidence was strongly positive over the period (AAPC 5.56%, 95% CI 1.38, 9.74) [Table 1]. All of this trend was due to the increasing rates 2008–2018, with no significant change in incidence 1990–2007. There was no significant difference in trend by age (0–49 vs. 50+).

## 4 DISCUSSION

Cancers of the breast, and most cancers of the female genital tract (cervix and corpus uteri, vulva) increase in incidence over the 30 year period (1990–2019) studied. As described in the previous study^7^ there was a decline in the numbers of cancer cases being diagnosed and treated in Harare in the years 2007–2009 during a period of political, economic and social challenges in Zimbabwe. Most departments of government central referral hospitals operated below capacity and some of them closed altogether, and the data from one of the major pathology laboratories were lost completely. As a result, there was a decrease in recorded incidence and the proportion of cases with morphological verification of diagnosis in these years. This can be seen as a dip in the curves of incidence over time. However, since this period was near the middle of the time considered, it does not much affect the overall trends over 30 years reported here. The exclusion of data from the 3 years in question did not alter any of the statistically significant findings. We therefore retained these data points in all presented analyses and figures.

For cancers of the breast, older age at first birth, reduced duration of breastfeeding, and lower parity are all associated with increasing breast cancer risks, as are low physical activity (increased sedentarism) and increasing obesity.

Fertility rates are declining across sub‐Saharan Africa, and total fertility rate in Zimbabwe decreased from 5.4 in 1988 to 3.7 in 2017.^16^ The Demographic and Health Surveys,^17^ estimates that the prevalence of women with a BMI > 25 kg/m^2^ has increased from 23% in 1994 in Zimbabwe, to 34.9% in 2015. On the other hand, the prevalence of insufficient physical activity in women in Zimbabwe is relatively low by global standards (~21% in 2022) and has decreased somewhat since 2000.^18^

The increased risk conferred by these risk factors is more marked in post‐menopausal women. Although there was no difference in trends in women in Harare aged 50+ compared with ages 15–49, there was indeed no increasing trend in incidence in successive generations of women aged less than 40.

Oral contraceptives and hormone replacement therapy (HRT) also increase risk of breast cancer. Between 1984 and 2015, the proportion of women who use injectable contraceptives has increased (from 1% to 10%) and the proportion of women who use oral contraceptives has almost doubled (from 23% to 41%).^19^ The increase in exposure to these hormonal agents might be expected to affect rates in pre‐menopausal women since the excess risk disappears quite rapidly in ex‐users.^20^

Cancer of the cervix uteri has been the most common cancer of women ever since the inception of the registry, and so it remains. The rates have increased at an average of 1% a year, reaching a peak ASR of 93.4 in 2005; since then, they have been relatively stable. The reasons for this trend and the resulting very high rates of disease are not immediately clear. The possible link to the epidemic of HIV/AIDS is complex. Cervix cancer is considered to be an AIDS‐defining condition. Women living with HIV (WLWH) have a higher risk of acquiring HPV, higher rates of HPV persistence, and faster progression to cervix cancer than women without HIV.^21^ WLWH have a 2–5 fold higher incidence of cervical precancerous lesions (high‐grade squamous intraepithelial lesion, HSIL) and a 4–6 fold higher incidence of cervix cancer compared to women without.^22^ Invasive cervix cancer is observed to occur 7–15 years earlier among WLWH compared with their HIV‐negative counterparts.^23^ However, the prevalence of HIV infection in Zimbabwe reached a maximum of around 23% among adults (15–49) in 1997, before falling to 18.4% in 2005 and 12.8% in 2019 (UNAIDS 2020),^24^ and availability and use of ART has been increasing since 2004; there is evidence that early ART initiation and sustained adherence is likely to reduce incidence and progression of CIN and ultimately the incidence of invasive cervix cancer in women living with HIV.^25^ The observation that there was no change in incidence in young women (20–34), the age group most affected by HIV/AIDS, suggests that the epidemic had little effect on population rates.

The observation that the increasing rates are more marked in generations of women born between 1950 and 1970 suggests other factors are in play. A rising risk of cervix cancer must imply an increase in prevalence of HPV infection, which likely reflects broader transmission patterns influenced by evolving sexual behaviors, mirroring patterns observed with other sexually transmitted infections. Additionally, gaps in vaccination coverage and uneven condom use could contribute to sustained transmission within key populations. No data are available on the HPV burden in the general population of Zimbabwe.^26^ However, studies of attendees at screening clinics in rural settings have shown relatively high prevalence of high‐risk HPV types (~15%) with higher prevalence in women positive for HIV.^27^, ^28^

In 2018, WHO adopted a global strategy to eliminate cervical cancer.^2^ This ambitious plan aims to achieve several key goals by 2030: vaccinating 90% of girls by age 15, ensuring 70% of women receive high‐performance screening tests at 35 and 45 years old, and treating 90% of women diagnosed with precancerous or cervical disease. WHO has developed cervical cancer screening guidelines adapted for WLWH.^29^

In high income countries, decreases in incidence have largely paralleled the introduction of effective screening programs based on cytology and have occurred despite high prevalence of persistent human papillomavirus (HPV) infection.^30^ The Ministry of Health and Child Care Zimbabwe introduced screening by visual inspection (VIA) in 2011. In order to make cervix cancer screening affordable to the majority of women in the country, VIA is offered for free in public hospitals.^31^ Yet, despite these efforts, the uptake of screening for cervix cancer in Zimbabwe is still quite low. In 2015, only 24% of women in Harare reported having had a cervical examination (88% of them within the last 5 years).^32^ Improving coverage of screening at ages 35 and 45, using recommended methods^29^ is a clear priority for cancer control in Zimbabwe.

HPV vaccination of girls aged 10–14 was introduced nationwide in Zimbabwe in 2018,^33^ with coverage of 91% (67% for two doses),^34^ but this had fallen to 40% post Covid. Vaccination of girls will have no effect on incidence rates of invasive cancer of the cervix for many years to come.

The incidence of ovarian cancer in the black population of Harare is relatively high—an average age standardised rate of 8.6 per 10^5^ over the 30‐year period, compared with the average of 5.1 per 10^5^ in sub‐Saharan Africa (and 6.7 per 10^5^ worldwide) in 2022.^1^ There has been no change in incidence over the 30‐year period, although rates have been noted to be increasing in other populations of sub‐Saharan Africa.^35^ Several factors have been associated with the risk of ovarian cancers. The risk decreases with lower parity, oral contraceptive use and tubal ligation,^36^ and fertility declines and increasing oral contraceptive use in Zimbabwe (noted earlier) might have been expected to lower incidence. It is possible that any decline in actual incidence has been offset by improved diagnosis, although there has been little change in the basis of diagnosis over the period (66% morphologically verified in 1990–94 compared with 60% in 2015–19).

The incidence of cancer of the corpus uteri in the black population of Harare is relatively high—the average age standardised rate of 9.2 per 10^5^ over the 30‐year period, is close to the global average of 8.4 per 10^5^ but considerably higher than the mean for sub‐Saharan Africa (3.4 per 10^5^) in 2022.^1^ Overweight/obesity are the most important environmental/lifestyle factors influencing risk,^37^ and, as noted earlier, the prevalence of women with a BMI > 25 kg/m^2^ has increased from 23% in 1994 in Zimbabwe, to 34.9% in 2015.^17^ This may, in part, explain the small (1% annual) increase in incidence in 1990–2019.

Cancer of the vulva appears to be relatively rare, although the average ASR of 2.1 per 10^5^ (and 3.3 per 10^5^ in the most recent decade) is high by global standards (ASR in 2022 = 0.83 per 10^5^) and is only matched by the estimated rates in other countries of South East Africa (Eswatini, Botswana, Mozambique).^1^ Like cancer of the cervix, invasive vulvar cancer is associated with oncogenic HPV, with a prevalence of infection greater than 70% among younger women (less than 60 years old), compared with less than 60% HPV‐positive tumours among older women.^38^, ^39^ This association most likely explains the correlation with high rates of cervix cancer in south‐eastern Africa, as well as the increasing incidence (as noted for cancers of the cervix). Increasing rates of vulvar cancer have been noted in other populations worldwide.^40^

In conclusion, these data from the Zimbabwe National Cancer Registry of Harare represent the only real information so far available on long term (30 years) trends of incidence of cancer in a population of sub‐Saharan Africa. Cancers of the breast and cervix are by far the most common forms of malignancy in women living in sub‐Saharan Africa (50% of all cancers^1^). Both have increased in incidence in Harare, as have cancers of the corpus uteri and vulva. These trends appear to be related to population‐level changes in lifestyles (fertility, obesity, contraception use), and exposure to infections with HIV and HPV, as published elsewhere. They also provide data on which to base projections of future burden, and hence a guide to priorities for cancer control (prevention, treatment and care).

## AUTHOR CONTRIBUTIONS

**Eric Chokunonga:** Data curation; investigation; funding acquisition; writing – review and editing. **Zvavahera Michael Chirenje:** Investigation; writing – review and editing; data curation. **Margaret Borok:** Validation; investigation; writing – review and editing; supervision. **Rudo Makunike‐Mutasa:** Investigation; writing – review and editing. **Ntokozo Ndlovu:** Investigation; writing – review and editing. **Justice Mudavanhu:** Investigation; writing – review and editing. **Biying Liu:** Project administration; writing – review and editing. **D. Maxwell Parkin:** Methodology; writing – review and editing; writing – original draft; formal analysis.

## FUNDING INFORMATION

We thank the Bloomberg Data for Health Initiative for the financial support via the Cancer Registration National Programme in Zimbabwe.

## CONFLICT OF INTEREST STATEMENT

The authors declare no conflicts of interest.

## ETHICS STATEMENT

Approval for our study was obtained from the Zimbabwe National Cancer Registry Advisory Committee. The study made use of routinely collected population‐level anonymised data. The study was performed in accordance with the Declaration of Helsinki.

## Data Availability

The data that support the findings of our study are available on request. All data requests will be evaluated by the AFCRN research committee. Details of the data application process are outlined on the AFCRN website http://afcrn.org/index.php/research/how-to-apply/76-research-collaborations. Further information is available from the corresponding author upon request.
